# Multiple Periocular Eccrine Hidrocystomas Mimicking Malignancy: A Clinicopathologic Diagnostic Challenge

**DOI:** 10.7759/cureus.106782

**Published:** 2026-04-10

**Authors:** Guillermo Roa Alvarez, Mario Shuchleib Cukiert, Evelyn I Figueroa Saavedra, Maria F Molina Hernandez, Luisa M Guerrero Escudero, Cristina Berumen-Glinz, Maria E Vega Memije

**Affiliations:** 1 Research Unit of Metabolic Diseases, Instituto Nacional de Ciencias Medicas y Nutrición Salvador Zubirán, Mexico City, MEX; 2 School of Medicine and Health Sciences, Tecnologico de Monterrey, Mexico City, MEX; 3 Department of Dermatology, Hospital General "Dr. Manuel Gea González", Mexico City, MEX; 4 Department of Medicine, Hospital General "Dr. Manuel Gea González", Mexico City, MEX; 5 Department of Dermatopathology, Hospital General "Dr. Manuel Gea Gonzalez", Mexico City, MEX; 6 Department of Dermatopathology, Hospital General "Dr. Manuel Gea González", Mexico City, MEX

**Keywords:** adnexal neoplasms, apocrine hidrocystoma, eccrine hidrocystoma, eyelid tumors, periocular lesions

## Abstract

Hidrocystomas are benign cystic tumors of sweat gland origin that may arise from eccrine or apocrine structures and frequently involve the periocular region. Their clinical resemblance to both benign and malignant adnexal lesions represents a diagnostic challenge. We report a case of multiple periocular eccrine hidrocystomas in a man in his 50s presenting with bilateral translucent cystic papules along the eyelid margins. Histopathologic evaluation revealed a well-circumscribed dermal cyst lined by a double layer of cuboidal epithelial cells without decapitation secretion, confirming eccrine differentiation. Given the potential for misdiagnosis as basal cell carcinoma or other adnexal neoplasms, accurate clinicopathologic correlation is essential to prevent unnecessary aggressive interventions. This case highlights distinguishing features, expands the differential diagnosis, and underscores the importance of histopathology in periocular cystic lesions.

## Introduction

Hidrocystomas are uncommon benign cystic lesions arising from sweat glands and are broadly classified into eccrine and apocrine subtypes based on histopathologic characteristics [1]. Although considered rare, they most frequently occur in the periocular region, where their translucent appearance and cystic morphology may mimic a variety of benign and malignant lesions. Apocrine hidrocystomas originate from apocrine glands associated with hair follicles, including the modified glands of Moll in the eyelids, whereas eccrine hidrocystomas are thought to arise from ductal obstruction of eccrine sweat glands [1,2].

Clinically, both subtypes present as translucent, dome-shaped papules or nodules. However, eccrine hidrocystomas more commonly present as multiple lesions with bilateral distribution, while apocrine hidrocystomas are typically solitary [2,3]. This distinction, although helpful, is not absolute and frequently necessitates histopathologic confirmation.

The periocular region represents a diagnostically challenging anatomical site due to the broad spectrum of lesions that may mimic hidrocystomas, including benign adnexal tumors, vascular lesions, and malignant neoplasms such as basal cell carcinoma [4]. Misdiagnosis may lead to overtreatment, including unnecessary surgical excision in cosmetically and functionally sensitive areas.

We present a case of multiple periocular eccrine hidrocystomas highlighting the diagnostic challenge, key distinguishing clinical features, and the importance of clinicopathologic correlation in avoiding unnecessary interventions.

## Case presentation

**Figure 1:**
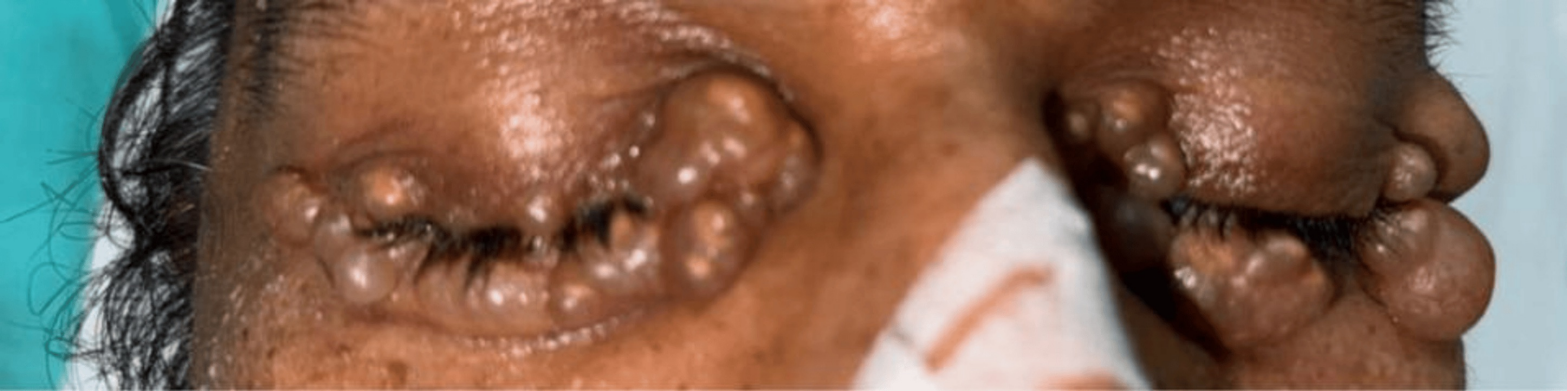
Multiple bilateral periocular translucent cystic papules consistent with eccrine hidrocystomas Multiple translucent, dome-shaped cystic papules clustered along the bilateral upper and lower eyelid margins, characteristic of periocular eccrine hidrocystomas, are noted.

A 54-year-old man presented with a several-year history of gradually progressive, asymptomatic periocular lesions. Dermatologic examination revealed multiple translucent to skin-colored, dome-shaped papules and cystic nodules symmetrically distributed along the upper and lower eyelid margins and medial canthi bilaterally (Figure 1). Lesions measured approximately 3-10 mm in diameter and were soft, fluctuant, and non-tender. No associated inflammation, ulceration, or discharge was observed. Visual acuity and ocular examination were unremarkable.

Key distinguishing clinical features included bilateral symmetric distribution, multiple translucent cystic papules, smooth surface without ulceration, absence of vascular changes, slow progressive growth, and lack of associated symptoms. These findings favored a benign cystic adnexal process.

The patient denied systemic symptoms, excessive sweating, or prior trauma or surgery in the affected area. There was no family history suggestive of genodermatoses or syndromic associations.

Given the multiplicity and periocular distribution, a broad differential diagnosis was considered, including syringomas, milia, epidermal inclusion cysts, apocrine hidrocystomas, trichilemmal cysts, vascular lesions, and cystic basal cell carcinoma [4-7]. The potential for malignancy and the risk of overtreatment in this region prompted histopathologic evaluation.

**Figure 2:**
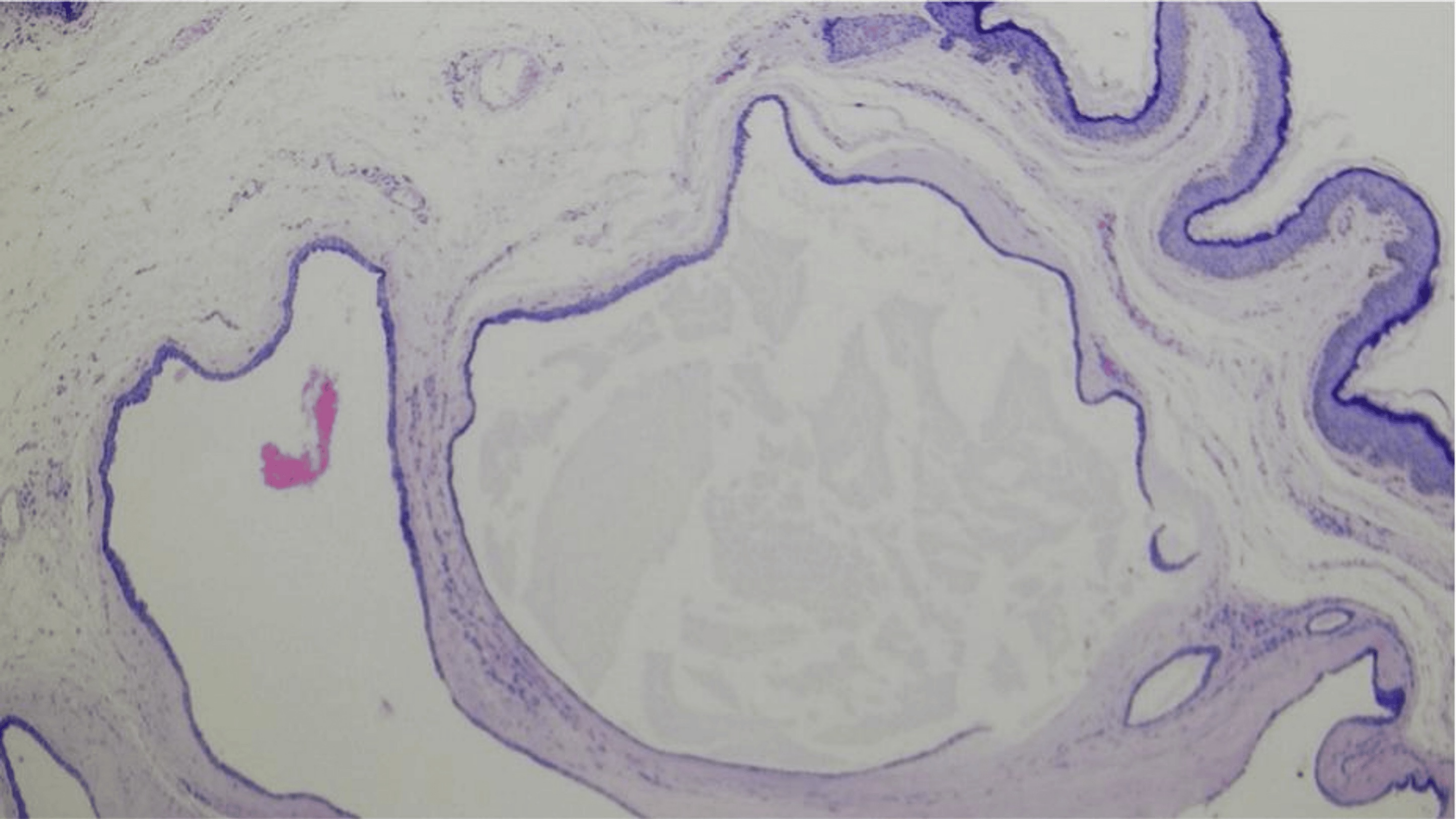
Histopathology of eccrine hidrocystoma showing a dermal cyst lined by cuboidal epithelium (H&E) Histopathologic photomicrograph (40X H&E) showing a well-circumscribed dermal cyst lined by a double layer of cuboidal epithelial cells without atypia or decapitation secretion, consistent with eccrine hidrocystoma.

Excisional biopsy of a representative lesion demonstrated a well-defined unilocular cyst within the dermis lined by a double layer of cuboidal epithelial cells without atypia or mitotic figures (Figure 2). The cyst lumen contained eosinophilic material. Notably, no decapitation secretion, papillary projections, or myoepithelial proliferation was identified, excluding apocrine differentiation [1,2]. There was no evidence of infiltrative growth or malignancy.

These findings confirmed the diagnosis of multiple periocular eccrine hidrocystomas.

## Discussion

Hidrocystomas represent benign cystic proliferations of sweat glands, with eccrine and apocrine variants demonstrating overlapping clinical presentations but distinct histologic features [1,2]. Eccrine hidrocystomas are believed to result from obstruction of eccrine ducts leading to retention cyst formation, and their enlargement in warm or humid environments supports this mechanism [3].

In contrast, apocrine hidrocystomas arise from proliferative changes in apocrine glands and demonstrate characteristic decapitation secretion histologically. They are more commonly solitary and may exhibit pigmentation due to lipofuscin accumulation, occasionally mimicking melanocytic lesions [2,8].

The presence of multiple periocular lesions, as seen in this case, strongly favors eccrine origin. However, multiple apocrine hidrocystomas, although rare, have been associated with genetic syndromes such as Schöpf-Schulz-Passarge and Goltz-Gorlin syndromes, as well as endocrine abnormalities, including prolactinomas [9,10]. The absence of systemic findings in this patient makes these associations unlikely.

The differential diagnosis of periocular cystic lesions is broad. Syringomas typically present as small, firm papules rather than cystic lesions. Epidermal inclusion cysts demonstrate keratinizing squamous epithelium histologically. Vascular lesions such as hemangiomas and lymphangiomas may appear translucent but show distinct clinical and imaging characteristics. Malignant entities, particularly basal cell carcinoma, may exhibit cystic features and represent the most clinically significant diagnostic pitfall [4-7].

Histopathologic examination remains the gold standard for diagnosis. The absence of decapitation secretion and the presence of a simple cuboidal epithelial lining are key features distinguishing eccrine from apocrine hidrocystomas [1,2].

Management is generally pursued for cosmetic reasons. Treatment options include surgical excision, electrodessication, laser therapy, and topical anticholinergic agents aimed at reducing eccrine secretion [11,12]. Recurrence is common, particularly in patients with multiple lesions, and long-term management may require repeated interventions.

This case emphasizes the importance of recognizing bilateral multiplicity and translucent cystic morphology as clinical clues while reinforcing the essential role of histopathology in excluding malignancy.

## Conclusions

Multiple periocular eccrine hidrocystomas represent a benign yet diagnostically challenging entity due to their clinical overlap with a broad spectrum of cystic and adnexal lesions, including malignant tumors such as basal cell carcinoma. Recognition of key clinical features, particularly multiplicity, bilateral distribution, and translucent cystic morphology, may suggest a benign sweat gland origin; however, these findings alone are insufficient to reliably exclude malignancy, especially in anatomically sensitive regions such as the eyelids. Histopathologic evaluation remains essential for definitive diagnosis, allowing accurate differentiation between eccrine and apocrine subtypes and exclusion of malignant adnexal neoplasms. The absence of decapitation secretion and the presence of a simple cuboidal epithelial lining are critical features supporting eccrine differentiation.

From a clinical standpoint, maintaining a broad differential diagnosis and emphasizing clinicopathologic correlation are fundamental to appropriate management. Failure to establish an accurate diagnosis may result in unnecessary invasive procedures, including aggressive surgical excision, with potential functional and cosmetic morbidity. Additionally, clinicians should remain aware of the rare association of multiple hidrocystomas with systemic or genetic conditions, which may warrant further evaluation in selected cases. Improved recognition of this entity can enhance diagnostic accuracy, guide therapeutic decision-making, and prevent overtreatment, while continued reporting may further define its clinical spectrum and optimal management strategies.
